# Punicalagin Attenuates Disturbed Flow-Induced Vascular Dysfunction by Inhibiting Force-Specific Activation of Smad1/5

**DOI:** 10.3389/fcell.2021.697539

**Published:** 2021-06-28

**Authors:** Gulinigaer Anwaier, Guan Lian, Gui-Zhi Ma, Wan-Li Shen, Chih-I Lee, Pei-Ling Lee, Zhan-Ying Chang, Yun-Xia Wang, Xiao-Yu Tian, Xiao-Li Gao, Jeng-Jiann Chiu, Rong Qi

**Affiliations:** ^1^Department of Pharmacology, School of Basic Medical Sciences, Peking University Health Science Center, Beijing, China; ^2^Key Laboratory of Molecular Cardiovascular Sciences, Ministry of Education, Peking University, Beijing, China; ^3^State Key Laboratory of Natural and Biomimetic Drugs, Peking University, Beijing, China; ^4^National Health Commission (NHC) Key Laboratory of Cardiovascular Molecular Biology and Regulatory Peptides, Peking University, Beijing, China; ^5^Beijing Key Laboratory of Molecular Pharmaceutics and New Drug Delivery Systems, Peking University, Beijing, China; ^6^College of Pharmacy, Xinjiang Medical University, Xinjiang, China; ^7^Xinjiang Key Laboratory of Active Components and Drug Release Technology of Natural Drugs, Xinjiang, China; ^8^School of Medical Laboratory Science and Biotechnology, College of Medical Science and Technology, Taipei Medical University, Taipei, Taiwan; ^9^Taipei Heart Institute, Taipei Medical University, Taipei, Taiwan; ^10^Institute of Cellular and System Medicine, National Health Research Institutes, Miaoli, Taiwan; ^11^Institute of Biomedical Engineering, National Tsing Hua University, Hsinchu, Taiwan; ^12^Institute of Polymer Science and Engineering, National Taiwan University, Taipei, Taiwan; ^13^School of Biomedical Sciences, Chinese University of Hong Kong, Hong Kong, China

**Keywords:** pomegranate, punicalagin, oscillatory shear stress, atherosclerosis, endothelial cell, Smads

## Abstract

**Background:**

Pathophysiological vascular remodeling in response to disturbed flow with low and oscillatory shear stress (OSS) plays important roles in atherosclerosis progression. Pomegranate extraction (PE) was reported having anti-atherogenic effects. However, whether it can exert a beneficial effect against disturbed flow-induced pathophysiological vascular remodeling to inhibit atherosclerosis remains unclear. The present study aims at investigating the anti-atherogenic effects of pomegranate peel polyphenols (PPP) extraction and its purified compound punicalagin (PU), as well as their protective effects on disturbed flow-induced vascular dysfunction and their underlying molecular mechanisms.

**Methods:**

The anti-atherogenic effects of PPP/PU were examined on low-density lipoprotein receptor knockout mice fed with a high fat diet. The vaso-protective effects of PPP/PU were examined in rat aortas using myograph assay. A combination of *in vivo* experiments on rats and *in vitro* flow system with human endothelial cells (ECs) was used to investigate the pharmacological actions of PPP/PU on EC dysfunction induced by disturbed flow. In addition, the effects of PPP/PU on vascular smooth muscle cell (VSMC) dysfunction were also examined.

**Results:**

PU is the effective component in PPP against atherosclerosis. PPP/PU evoked endothelium-dependent relaxation in rat aortas. PPP/PU inhibited the activation of Smad1/5 in the EC layers at post-stenotic regions of rat aortas exposed to disturbed flow with OSS. PPP/PU suppressed OSS-induced expression of cell cycle regulatory and pro-inflammatory genes in ECs. Moreover, PPP/PU inhibited inflammation-induced VSMC dysfunction.

**Conclusion:**

PPP/PU protect against OSS-induced vascular remodeling through inhibiting force-specific activation of Smad1/5 in ECs and this mechanism contributes to their anti-atherogenic effects.

## Introduction

Atherosclerosis is a chronic inflammatory vascular disorder highly associated with endothelial cell (EC) dysfunction, vascular smooth muscle cell (VSMC) proliferation and migration, inflammatory monocyte infiltration, lipid deposition, and vascular wall remodeling (Gimbrone and Garcia-Cardena, 2016). The non-random distribution of atherosclerotic lesions is related to different patterns of blood flow and hemodynamic forces acting on the vascular wall. As an important signal transduction medium between blood flow and arterial wall, vascular ECs are constantly exposed to different flow patterns and shear stresses, including disturbed flow with low and oscillatory shear stress (OSS) and pulsatile flow with relatively high shear stress (PSS), leading to distinct impacts on the vascular wall (Zhou et al., 2014). Plaques preferentially occur at arterial branches and curvatures where the local flow is disturbed with OSS (Chiu et al., 2009). By contrast, arterial regions exposed to pulsatile flow with PSS are relatively lesion-free (Sorescu et al., 2003). Pulsatile flow with PSS generally is anti-inflammatory and anti-atherogenic, whereas disturbed flow with OSS promotes the formation and progression of atherosclerosis (Gimbrone and Garcia-Cardena, 2016). Our previous studies and others have shown that ECs are capable of perceiving OSS as a mechanical signal to induce force-specific activation of bone morphogenetic protein receptor (BMPR)-associated Smad1/5, leading to upregulation of cyclin A and downregulation of p21 and p27, thereby increasing EC cell cycle progression and proliferation (Sorescu et al., 2003; Chang et al., 2007; Zhou et al., 2012, 2013). OSS-activated Smad1/5 can further promote the activation of nuclear factor-κB (NF-κB) pathways and release of pro-inflammatory cytokines interleukin-1β (IL-1β) and tumor necrosis factor-α (TNF-α) to aggravate EC injury (Zhou et al., 2012, 2013). OSS-induced pro-inflammatory responses in ECs can elicit chemotaxis and adhesion of monocytes to the EC layers mediated by intercellular adhesion molecule-1 (ICAM-1), vascular cell adhesion molecule-1 (VCAM-1), and monocyte chemotactic protein-1 (MCP-1), which further promotes atherosclerosis progression (Chiu et al., 2004). On the other hand, the injured ECs produce pro-inflammatory cytokines such as TNF-α to promote phenotypic modulation, proliferation and migration of VSMCs to further aggravate atherosclerosis (Sorescu et al., 2003). Taken together, all of these sequential events suggest that force-specific activation of Smads may be a promising hemodynamic-based molecular target for intervention against disturbed flow-associated vascular disorders, such as atherosclerosis.

Pomegranate peel is rich in polyphenols with the main component of punicalagin. Pomegranate extraction (PE) was reported to exert anti-atherogenic effects *via* lowering circulating levels of low-density lipoprotein and formation of macrophage-derived foam cells (Al-Jarallah et al., 2013; Rosenblat et al., 2013; Atrahimovich et al., 2016, 2018). Prolonged PE supplementation inhibited OSS-related atherosclerosis by upregulating endothelial nitric oxide synthase (eNOS) expression and modulating oxidation-sensitive gene expression in ECs (de Nigris et al., 2007). However, whether the extraction of pomegranate peel polyphenols (PPP) and its purified compound punicalagin (PU) exert ameliorative effects on OSS-induced vascular dysfunction and hence protect against atherosclerosis remain unclear.

In the present study, we demonstrated for the first time that PU is the bioactive anti-atherogenic constituent of PPP. We further investigated the roles of PPP and PU in OSS-induced EC dysfunctions and inflammation-related VSMC pathophysiological responses *in vitro* and *in vivo*. Our results showed that PPP and PU exert protective effects on the arterial wall and atherosclerosis by attenuating disturbed flow/inflammation-induced vascular dysfunction, mainly through suppressing force-specific activation of Smad1/5 in ECs. Thus, our study provides new information to indicate that PPP and PU may have great potential to be developed as new therapeutic components for the treatment of atherosclerosis.

## Materials and Methods

### Materials

PPP (containing 42.08% punicalagin) and PU (containing 90.6% punicalagin) (Supplementary Table 1) were provided by Prof. Xiao-Li Gao from Xinjiang Medical University, China. Extraction methods and HPLC analysis of PPP/PU were described in Supplementary Data (Supplementary Figure 1). Punicalagin reference substance (purity 98%) was purchased from Yuanye Biological Technology Co., Ltd., (Shanghai, China). Serum triglyceride (TG) and total cholesterol (TC) kits were purchased from Biosino Bio-technology and Science Inc. (Nanjing, China). TNF-α was purchased from Peprotech (Rocky Hill, United States). Fetal bovine serum (FBS, Gibco), Dulbecco’s Modified Eagle’s Medium (DMEM) and medium 199 (M199, Gibco, Grand Island, NY, United States) were from GIBCO (Grand Island, United States). Rabbit anti-phospho-Smad1/5 (#AB3848-I), rabbit anti-von Willebrand Factor (#AB7356) were purchased from Millipore (Massachusetts, United States). Rabbit anti-Smad1/5 (#sc6031R) was from Santa Cruz Biotechnology (California, United States). Rabbit anti-Mac-2 (#ab217760), anti-VCAM-1 (#ab134047), anti-α-SMA (#ab14106), and mouse anti-α-SMA (#ab7817) were purchased from Abcam (Cambridge, United Kingdom). Rabbit anti-ICAM-1 (#4915), rabbit anti-phospho-Rb (#9308), mouse anti-Rb (#9309), mouse anti-Cyclin A (#4656) and mouse anti-Ki-67 (#9449) were purchased from Cell signaling (Massachusetts, United States). Rabbit anti-E-selectin (#GTX54691) was purchased from GeneTex (Irvine, CA). Mouse anti-GAPDH antibody was purchased from Santa Cruz (Dallas, United States). The HRP-conjugated secondary antibodies, affinity-purified mouse anti-rabbit IgG, and rabbit anti-mouse IgG were purchased from Sigma (St. Louis, MO, United States). All other chemical agents were purchased from Sigma Aldrich (St. Louis, MO, United States) unless otherwise noted.

### Anti-atherogenic Studies of PPP/PU

Healthy 6-week-old male low-density lipoprotein receptor knockout (LDLR^–/–^) mice (Jackson Laboratory, United States) were housed with free access to water and standard laboratory chow diet. Atherosclerosis was induced by feeding LDLR^–/–^ mice with a high fat diet (HFD) containing 20% lard and 0.5% cholesterol for 12 weeks. LDLR^–/–^ mice were randomly assigned to the treatment groups. Vehicle group mice were administrated with phosphate-buffered saline (PBS) by gavage. In drug treatment group, PPP or PU was dissolved in PBS and given daily to mice by gavage at different doses. After 12 weeks, all mice were euthanized with an overdose of sodium pentobarbital. The blood samples were taken from the mice after 12-h fasting and centrifuged (Eppendorf 5418R, Eppendorf Corporation, Hamburg, Germany) at 1,400 × *g* for 10 min at 4°C. And the plasma was taken for measure of plasma triglycerides (TG) and total cholesterol (TC) according to manufacturer’s instructions. The aortas were isolated for *en face* immunostaining and aortic roots were used for Oil Red O and immunohistochemical staining. Briefly, mouse aortas were fixed with 4% paraformaldehyde for 2 h and dehydrated in 20% sucrose solution overnight and aortic span sections were stained by 0.5% Oil Red O for detecting lipid deposition in plaques. Positive areas of Oil Red O staining in the lesions were quantified using Image ProPlus 6.0 image analysis software. Mouse aortic roots were cut into serial frozen sections containing 300 cross-sections in 7 μm thickness, and ten cross-sections obtained from an interval of 30 sections were used for Oil Red O staining to analyze plaque areas. Atherosclerosis sections were blinded per individual to avoid the bias. Representative images of immunohistochemical staining of Mac-2 indicated the number of macrophages in atherosclerotic lesions.

### Endothelium-Dependent Vaso-Protective Assay

Effects of PPP and PU on eNOS activity were detected by myograph. Briefly, Sprague Dawley male rats were euthanized by an overdose of carbon dioxide. The aorta was dissected and excised quickly and placed in ice-cold physiological saline solution (PSS) containing (in mmol/l) 119 NaCl, 4.7 KCl, 25 NaHCO_3_, 1.17 KH_2_PO_4_, 1.17 MgSO_4_, 1.6 CaCl, and 5.5 dextrose, gassed by 95% O_2_–5% CO_2_. The aorta was then cut into 3 mm vessel rings and the EC layer was stripped off in -endo group. The vessel rings were incubated with PPP or PU at different concentrations (from 1 to 75 μg/mL for PPP and 1–50 μg/mL for PU) in the presence and absence of nitric oxide synthase inhibitor L-N(G)-nitroarginine methyl ester (L-NAME, 100 μM). Vessel viability was detected by the stimulation of phenylephrine (PE) and acetylcholine. PE stimulation would constrict the integrated vessel ring and acetylcholine stimulation could relax the PE-constricted vessel. When the EC layer was removed or treated with L-NAME, acetylcholine could not make the constricted vessel ring relaxation.

### Aortic Stenosis Studies in Rats

Aortic stenosis was induced in the rat by using a U-shaped titanium clip to constrict its abdominal aorta for 2 weeks as described (Miao et al., 2005; Zhou et al., 2012, 2013). Briefly, following anesthetization with isoflurane, the rat was laid supine and a lower midline abdomen incision was made. Thereafter, the part of the intestine was gently lifted out of abdomen and kept moist with saline throughout the surgical procedure. The aorta, left and right common iliac artery were exposed and the accompanying vein was carefully separated. The clip was held with a pair of forceps and placed around the isolated segment (approximate 1 cm from the arterial bifurcation) to partially constrict rat’s abdominal aorta. The extent of clipping was controlled by placing a stopper of given size between the two arms of the forceps. Our previous study using ultrasonography indicated that placement of the U-clip resulted in a 65% constriction of the aorta diameter, which induced an accelerated forward laminar flow in the constricted region, followed by a pronounced oscillating flow with the existence of retrograde velocities downstream in the region of poststenotic dilatation (Zhou et al., 2012). The flow patterns and wall shear stress distributions in the constricted rat abdominal aorta were further characterized by computational fluid dynamic modeling using the Comsol Multiphysics software, which confirmed the existence of recirculation eddies with retrograde velocities downstream to the constricted sites (Zhou et al., 2012). PPP (750 mg/kg/day) or PU (500 mg/kg/day) was given daily by gavage 3 days before operation and the daily treatment lasted for additional 2 weeks. All rats were sacrificed by the end of treatment and aortas were perfusion-fixed with 4% paraformaldehyde at 120 mmHg. The fixed aortas were embedded in paraffin blocks for immunohistochemical studies.

### Immunohistochemical Staining

Briefly, rats were euthanized with CO_2_ and transcardially perfused with 150 mL of saline, followed by 500 mL of 10% (vol/vol) neutral-buffered zinc-formalin (Thermo Fisher Scientific). After perfusion, the aortas were harvested and postfixed in the fixative solution for 1 h, and then subjected to immunohistochemical staining. Tissues were washed in Tris-buffered saline (TBS) buffer, and the adventitia was carefully removed. The aorta was then longitudinally cut open with microdissecting scissors and pinned flatly on a black wax dissection pan. The luminal surface of the aorta was immediately blocked with 4% (vol/vol) FBS for 1 h, followed by incubation with the designed primary antibodies, including rabbit anti-phospho-Smad1/5 (1:100), rabbit anti-von Willebrand Factor (1:100) and mouse anti-α-SMA (1:100) at 4°C overnight. Dylight 594-conjugated anti-goat IgG (1:300; Jackson ImmunoResearch) and Alexa Fluor 488-conjugated goat anti-rabbit IgG (1:300; Invitrogen) were used as secondary antibodies. Samples were counterstained with 4′,6′-diamidino-2-phenylindole (DAPI) to show cell nuclei, rinsed three times in TBS, mounted with glycerol/PBS (1:1), and photographed with a Leica TCS SP5 confocal microscope.

### Isolation and Culture of Primary ECs

ECs were isolated from fresh human umbilical cords with collagenase perfusion technique (Gimbrone, 1976). The cell pellets were resuspended in a culture medium consisting of medium 199 (M199, Gibco, Grand Island, NY, United States) supplemented with 20% fetal bovine serum (FBS, Gibco) and 1% penicillin/streptomycin (Gibco). ECs were grown in Petri dishes for 3 days and then seeded onto glass slides (75 by 38 mm, Corning, NY, United States) pre-coated with fibronectin (Sigma) to reach confluence. The culture medium was then replaced by the identical medium that contained only 2% FBS, and the cells were incubated for 24 h before use.

### Oscillatory Flow Apparatus

The cultured ECs were subjected to shear stress in a parallel-plate flow chamber, as previously described (Zhou et al., 2012, 2013). The chamber containing the cell-seeded glass slide fastened with the gasket was connected to a perfusion loop system, kept in a constant-temperature controlled enclosure, and maintained at pH 7.4 by continuous gassing with a humidified mixture of 5% CO_2_ in air. The fluid shear stress on the ECs can be estimated as τ = 6μQ/wh^2^, where τ is shear stress, Q is the flow rate, and μ is dynamic viscosity of the perfusate. The oscillatory flow is composed of a low level of mean flow with shear stress at 0.5 dynes/cm^2^ supplied by a hydrostatic flow system and the superimposition of a sinusoidal oscillation using a piston pump with a frequency of 1 Hz and a peak-to-peak amplitude of 4 dynes/cm^2^. In the *in vitro* experiments, ECs were pre-treated with PPP (50 μg/mL) or PU (50 μg/mL) for 30 min and then subjected to OSS (0.5 ± 4 dynes/cm^2^) in a parallel-plate flow chamber for 4 or 24 h, or stimulated with BMP (100 ng/mL) for 30 min or TNF-α (100 U/mL) for 4 h in the presence of PPP or PU.

### Immunofluorescence Assay (IFA)

ECs were seeded on coverslips in the culture plate wells and subjected to OSS. IFA was performed using antibodies against ki67 (Abcam), as described (Wang et al., 2016). The coverslips were mounted onto the slides using Fluoromount-G clear mounting medium containing DAPI (Southern Biotech, Birmingham, AL, United States). The fluorescence signals were observed *via* fluorescence microscopy (Nikon ECLIPSE Ti), and images were taken using NIS-Elements F software (Nikon).

### Isolation and Culture of Primary VSMCs

Primary VSMCs were obtained from rat thoracic arteries, as described (Wang et al., 2015; Shen et al., 2019). Briefly, male SD rats weighing about 100 g were anesthetized and thoracic arteries were carefully excised, and the surrounding perivascular adipose tissues and connective tissues were trimmed off. The arteries were washed in 0.01 M PBS containing 100 g/mL streptomycin and 100 IU/mL penicillin. Arterial ectoderm was removed and sliced with an ophthalmic scissor, and the vascular endothelium was scratched gently using curved dissection forceps. Vascular tissues were washed and cut into small pieces. They were placed at the bottom of a 100 mm culture dish filled with 1 mL DMEM with 10% FBS, 100 g/mL streptomycin and 100 IU/mL penicillin and incubated at 37°C for 6 h until they all stick to the bottom of the dish. Thereafter, culture medium was replenished every 4 days. The cells between passages 4 and 7 were used.

### VSMC Migration Assay

The wound healing assay was performed to test migratory capability of VSMCs. Briefly, VSMCs were seeded into 24-well tissue culture plates. The cells were incubated in DMEM supplemented with 0.5% FBS for 24 h to reach 70–80% confluence. The cell layer was scratched gently with a new 200 μL pipette tip across the center of the well. The cells were washed three times in 1 × PBS to remove the detached cells and incubated in DMEM supplemented with 0.5% FBS. The cells were treated with TNF-α (100 U/mL) in the absence and presence of PPP or PU for additional 24 h, and then washed twice in 1 × PBS and finally stained with 1% crystal violet in 2% ethanol for 30 min. Photos were taken for the stained monolayer on a microscope. Multiple views of each well were documented, and each experimental group was repeated 5 times.

## Western Blot Analysis

The cells were lysed with a buffer containing 1% NP-40, 0.5% sodium deoxycholate, 0.1% SDS, and a protease inhibitor mixture (PMSF, aprotinin, and sodium orthovanadate). The total cell lysate (100 μg of protein) was separated by SDS-polyacrylamide gel electrophoresis (PAGE) (12% running, 4% stacking) and analyzed using the designated antibodies and detected by Western-Light chemilumi-nescent detection system (Applied Biosystems, Foster City, CA).

### RNA Isolation and Quantitative Real-Time PCR (RT-PCR)

Total RNA was extracted using Trizol reagent (Invitrogen, United States) and the first-strand cDNA was generated using an RT kit (Invitrogen, United States). Quantitative real-time PCR was performed using primers shown in Supplementary Table 2. Amplifications were performed using an Opticon-Continuous Fluorescence Detection System (MJ Research) with Eva Green fluorescence dye (Molecular Probes, Eugene, United States). All samples were quantitated by using the comparative CT method for relative quantitation of gene expression, normalized to GAPDH levels.

### Statistical Analysis

All statistical analyses were performed using GraphPad Prism for Windows (Version 4, San Diego, CA, United States). Values were expressed as mean ± standard error of the mean (SEM). All data sets were tested for normal distribution. For normally distributed data, unpaired *t*-test, one-way ANOVA with Tukey post-test or paired *t*-test were used as most appropriate. All results were considered significantly as *p* < 0.05.

## Results

### The Anti-atherogenic Effect of PPP

**FIGURE 1:**
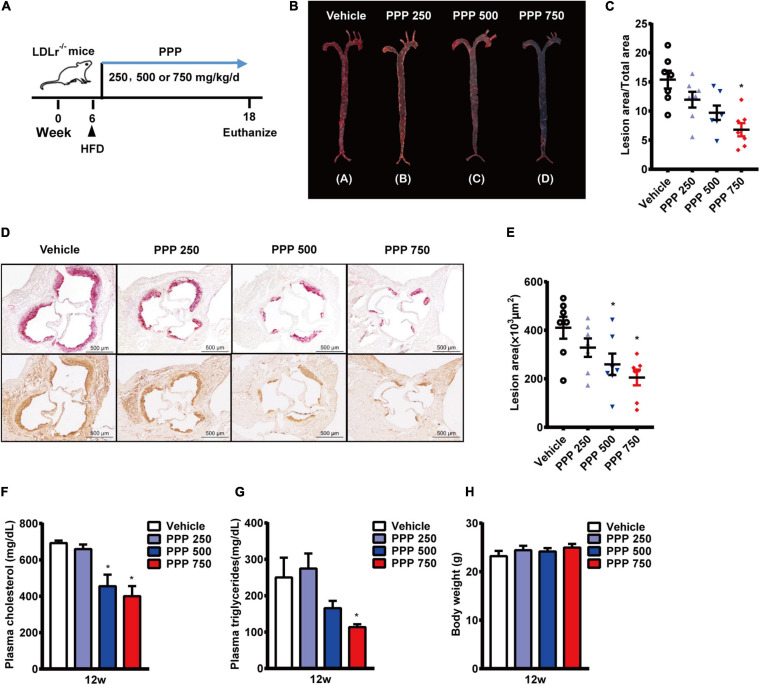
Anti-atherogenic effects of PPP on LDLR^–/–^ mice fed with HFD. **(A)** Schematic diagram of the experiments; **(B,C)** representative images of Oil Red O staining of mouse aortas **(B)** and quantitative results **(C)**; **(D,E)** representative images of Oil red O staining of mouse aortic outflow tract (upper) and immunostaining of Mac-2 (bottom); **(F,G)** plasma TC **(F)** and TG **(G)** levels after 12-week HFD feeding; **(H)** Body weights of the mice. PPP 250, PPP 500, and PPP 750 represent PPP at the doses of 250, 500, and 750 mg/kg/day, respectively, including *n* = 6 biological replicates. *P < 0.05 vs. Vehicle.

LDLR^–/–^ mice in 6 weeks old were fed with a HFD and treated daily with PPP for 12 weeks (Figure 1A). The *En face* immunostaining revealed that treatment with PPP at 750 mg/kg reduced the areas of Oil Red O-stained plaques by 56% in mouse aortas, whereas PPP at 250 mg/kg and 500 mg/kg decreased the plaque areas by 23 and 37%, respectively. These data indicate that PPP exerts anti-atherogenic effects *in vivo* in a dose-dependent manner (Figures 1B,C).

The lesion area of the aortic root is an important parameter of aortic stenosis and correlates with the severity of atherosclerotic plaques. Thus, plaque areas were further measured in cross-sections of aortic roots. The Oil Red O-staining results showed that treatment with PPP at 750 mg/kg reduced the plaque area by 49% compared to the vehicle control, and plaque area in the aortic roots was also reduced in the other two PPP-treated groups, with 500 mg/kg being more effective than 250 mg/kg (Figures 1D,E). Immmunohistochemical staining of the aortic roots showed dramatic reduction in macrophage content stained by Mac-2 in the lesion areas from the PPP-treated LDLR^–/–^ mice, indicating that PPP alleviated infiltration of inflammatory macrophages in the plaques (Figure 1D).

Disorder of plasma lipid metabolism has been deemed to play an important role in the progression of atherosclerosis. Treatment of LDLR^–/–^ mice with 750 mg/kg or 500 mg/kg PPP significantly lowered plasma levels of TC and TG (Figures 1F,G). No difference was observed in body weight among all groups (Figure 1).

### PU Is an Active Anti-atherogenic Compound in PPP

**FIGURE 2:**
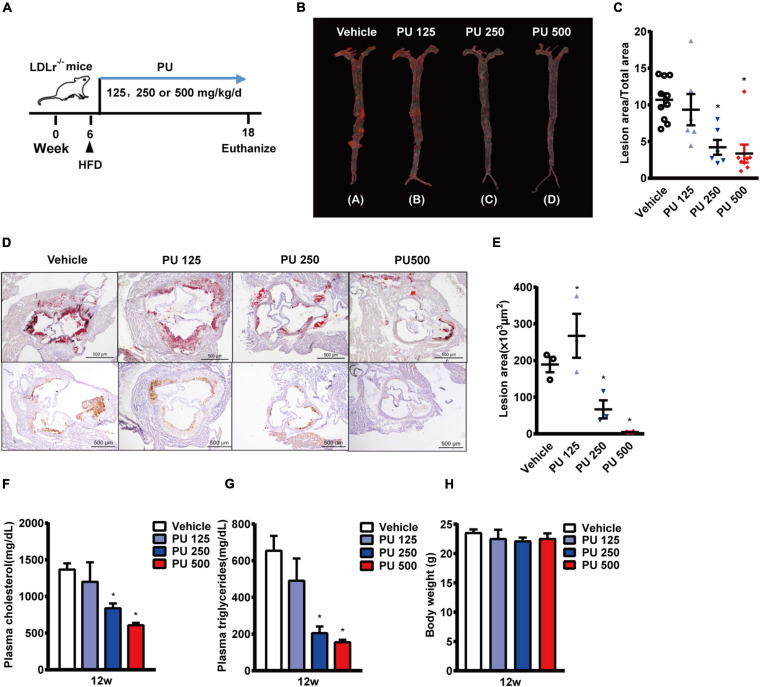
Anti-atherogenic effects of PU on LDLR^–/–^ mice fed with HFD. **(A)** Schematic diagram of the experiments; **(B,C)** representative images of Oil Red O staining of mouse aortas **(B)** and quantitative results **(C)**; **(D,E)** representative images of Oil red O staining of mouse aortic outflow tract (upper) and immunostaining of Mac-2 (bottom); **(F,G)**. plasma TC **(F)** and TG **(G)** levels after feeding HFD for 12 weeks; **(H)** Body weights of the mice. PU 125, PU 250, and PU 500 represent PU at the doses of 125, 250, and 500 mg/kg/day, respectively, including *n* = 5–10 biological replicates. *P < 0.05 vs. Vehicle.

Since PU is the major bioactive compound in PPP, we investigated whether PU treatment produces the similar protective effects on atherosclerosis as PPP. PU was administered daily to LDLR^–/–^ mice on HFD for 12 weeks (Figure 2A). The results showed that treatment with PU at 500 or 250 mg/kg reduced Oil Red O-stained atherosclerotic plaque areas in mouse aortas by 57 and 42%, respectively (Figures 2B,C). The Oil Red O staining of the plaque areas in aortic roots showed that PU treatment also decreased the lesion areas by 75 and 60%, respectively (Figures 2D,E). The number of infiltrated macrophages in aortic roots was reduced by PU (Figure 2D), and this effect was similar to that of PPP. In addition, PU treatment lowered plasma concentrations of TC and TG in HFD-fed LDLR^–/–^ mice (Figures 2F,G) without affecting body weight (Figure 2H). These results indicate that PU is the major anti-atherogenic component in PPP.

Next, we examined whether PPP and PU could inhibit macrophages to uptake lipids and their transformation into foam cells. The Oil red O-staining results showed that both PPP and PU reduced ox-LDL uptake into macrophages and thus inhibited foam cell formation (Supplementary Figure 2A). Meanwhile, PPP and PU decreased the lipopolysaccharide (LPS, 1 μg/mL)-induced expression of pro-inflammatory cytokines or chemokines including IL-1β, IL-6, TNF-α, and MCP-1, and inducible nitric oxide synthase (iNOS) (Supplementary Figures 2B–F). The results showed that PPP and PU inhibited macrophage-directed foam cell formation and further blocked the inflammatory cascade of macrophages to retard atherosclerosis progression, which were in agreement with the results of previous reports (Kaplan et al., 2001; Rosenblat et al., 2013; Aharoni et al., 2015).

### PPP and PU Exert Endothelium-Dependent Vaso-Protective Effects

**FIGURE 3:**
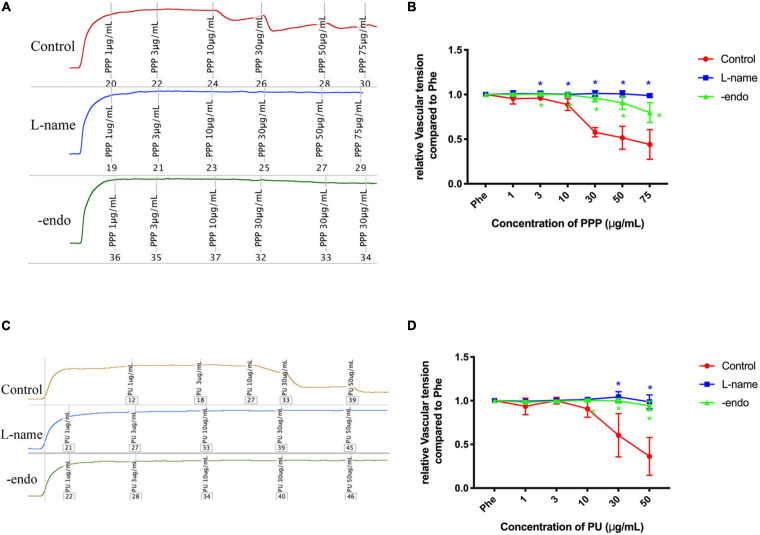
Endothelium-dependent vaso-protective effects of PPP and PU. A-D. Endothelium-dependent vaso-protective effects of PPP **(A)** and PU **(C)** and the quantitative results [**(B,D)**, respectively], including *n* = 4–6 biological replicates. *P < 0.05 vs. Control.

Then we examined whether PPP and PU can exert atheroprotective effects beyond the above effects but through improving vascular function. It is known that ECs are exposed to regular laminar shear stress in the normal physiological condition, which stimulates the release of nitric oxide (NO) by the sustained activation of eNOS (Buga et al., 1991). However, eNOS activity is reduced at sites of perturbed shear stress with OSS (Wang et al., 2019). There was a study showing that pomegranate juice enhances the biological actions of NO (Ignarro et al., 2006). Therefore, we investigated the effects of PPP and PU on the vascular reactivity. Firstly, the aorta vessel rings were incubated with PPP or PU at the designated concentrations with or without eNOS inhibitor L-NAME. Myograph system was used to detect the relaxation on pre-constricted vessel rings with endothelium (control) or without endothelium (-endo). The results showed that PPP and PU had a relaxation effect on PE-induced vasoconstriction, and this effect was abolished when the aortas were treated with L-NAME or the endothelia on the aortas were stripped off, demonstrating that the vasodilation effects of PPP and PU are endothelium-dependent (Figures 3A–D). These results indicate that PPP and PU may exert atheroprotective effects through improvement of endothelial function.

### PPP and PU Inhibit Activation of Smad1/5 in Vascular Endothelium Induced by Disturbed Flow *in vivo*

**FIGURE 4:**
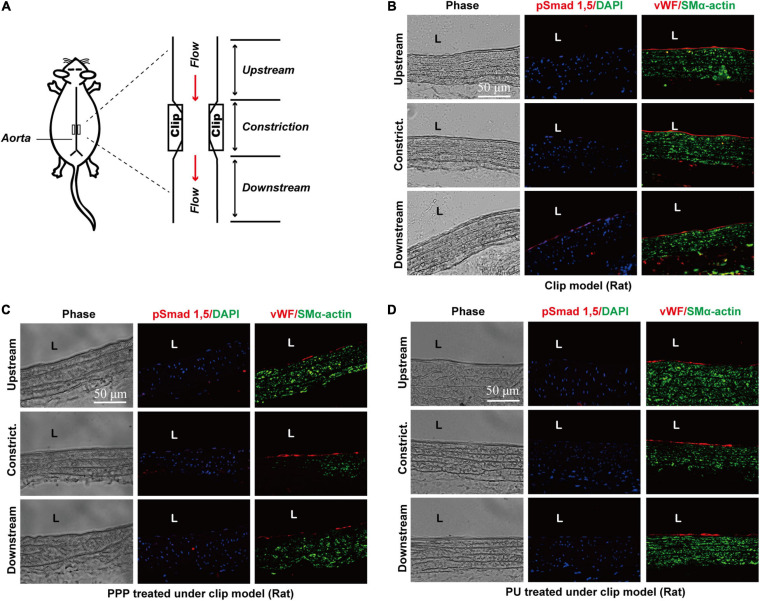
PPP and PU inhibit force-specific activation of Smad1/5 in ECs induced by disturbed flow *in vivo*. A rat model with abdominal aortic stenosis was established by partially constricting rat abdominal aorta using a U-clip **(A)**. Protein expressions of phospho-Smad1/5/vWF/SMα-actin in the upstream, middle point, and downstream areas of the constricted sites were detected by immunofluorescence staining. **(B)** saline treatment group, **(C)** PPP treatment group, and **(D)** PU treatment group (five independent experiments performed).

Our previous studies demonstrated that disturbed flow can induce force-specific activation of phospho-Smad1/5 in the post-stenotic sites, where the local flow is disturbed with OSS (Zhou et al., 2012). Thus, we examined whether PPP and PU could modulate this force-specific activation of Smad1/5 in post-stenotic sites *in vivo*. Rat abdominal aorta was subjected to a constriction by using a U-clip (Figure 4A), which can produce a disturbed flow region downstream to the constricted site, as described (Zhou et al., 2012, 2013). Either PPP or PU was administrated by gavage daily to the rats. Immunohistochemical examination of serial sections of the constricted aortas showed that the post-stenotic sites exhibited a high detection level of phospho-Smad1/5 in the luminal EC layer. By contrast, there was virtually no detectable staining of phospho-Smad1/5 in the upstream and middle point of constriction (Figure 4B). Treatment with PPP or PU dramatically decreased the elevation of phospho-Smad1/5 induced by disturbed flow at post-stenotic sites (Figures 4C,D). These results indicate that PPP and PU are effective in inhibiting force-specific activation of Smad1/5 in ECs induced by disturbed flow *in vivo*.

### PPP and PU Inhibit OSS-Induced Smad1/5 Phosphorylation in ECs *in vitro*

**FIGURE 5:**
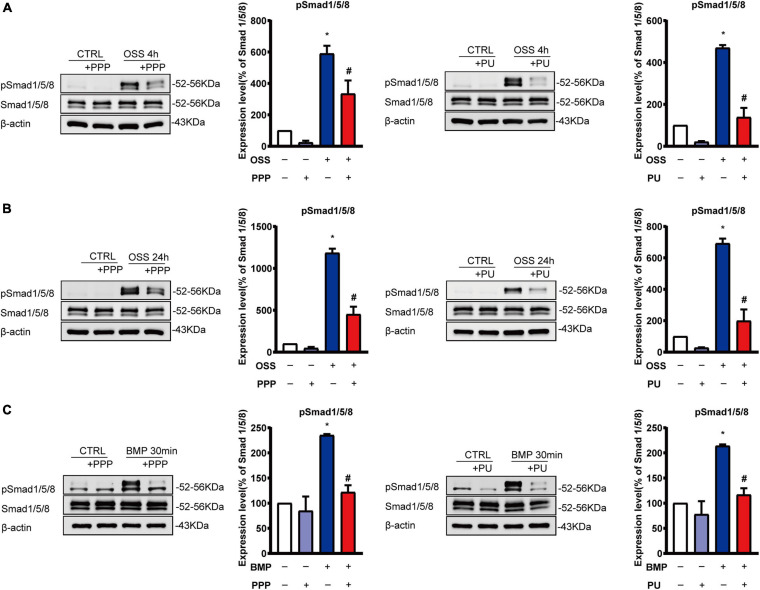
PPP and PU inhibit Smad1/5 activation in ECs induced by OSS or BMP. **(A–C)** Western blot analysis of protein expression of the indicated molecules in ECs induced by OSS for 4 h **(A)** or 24 h **(B)** and BMP for 30 min **(C)**. Blot is a representative of five independent experiments. *P < 0.05 vs. Vehicle, ^#^*P* < 0.05 vs. Model.

We next further tested the effects of PPP or PU on Smad1/5 activation in ECs in response to disturbed flow with OSS. Application of OSS to ECs induced a sustained phosphorylation of Smad1/5 in ECs over the 24-h period. The phosphorylation of Smad1/5 in ECs was induced rapidly (4 h) by OSS and remained elevated after 24 h, as compared with static control ECs. This OSS-induced phosphorylation of Smad1/5 in ECs was normalized to the basal level after PPP or PU treatment (Figures 5A,B).

Previous studies showed that OSS-induced activation of Smad1/5 in ECs is specifically through the activation of BMPRs (Zhou et al., 2012, 2013). Thus, we treated ECs with BMP for 30 min to active BMPR and its downstream Smads as a control experiment to explore the effects of PPP and PU on the BMP-elicited signaling in ECs. The results showed that PPP and PU significantly suppressed BMP-induced phosphorylation of Smad1/5 in ECs (Figure 5C).

### PPP and PU Inhibit OSS-Induced Pro-inflammatory Responses of ECs

**FIGURE 6:**
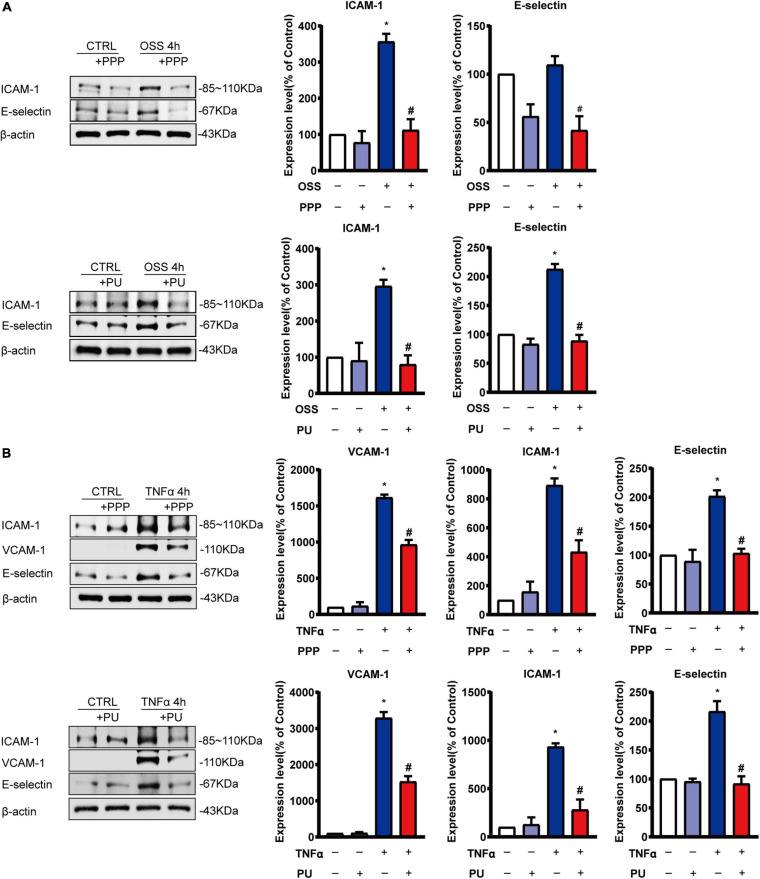
PPP and PU inhibit pro-inflammatory responses of ECs induced by OSS or TNF-α. **(A,B)** Western blot analysis and quantitative results of the effects of PPP and PU on OSS—**(A)** or TNF-α **(B)**—induced pro-inflammatory responses of ECs. Blot is a representative of five independent experiments. *P < 0.05 vs. Vehicle, ^#^*P* < 0.05 vs. Model.

EC inflammation is the early event in the pathogenesis of atherosclerosis, and OSS can activate EC pro-inflammatory responses by triggering the release of signaling molecules such as TNF-α to promote atherosclerosis progression (Zeiher et al., 1991; Sun et al., 2019). Thus, ECs were stimulated with OSS for 4 h or TNF-α for 4 h to stimulate the pro-inflammatory conditions of ECs and the effects of PPP or PU were examined. As expected, both OSS and TNF-α augmented the expressions of ICAM-1, VCAM-1 and E-selectin at both mRNA and protein levels in ECs (Supplementary Figure 3 and Figure 6). Pre-treatment with PPP or PU abolished either OSS or TNF-α–induced protein expression of these molecules (Figures 6A,B). Likewise, mRNA expression of these genes was also reversed by PPP or PU treatment (Supplementary Figures 3A,B). These results indicate that PPP and PU exert inhibitory effects on OSS-induced pro-inflammatory responses in ECs.

### PPP and PU Inhibit OSS-Induced Proliferation of ECs

**FIGURE 7:**
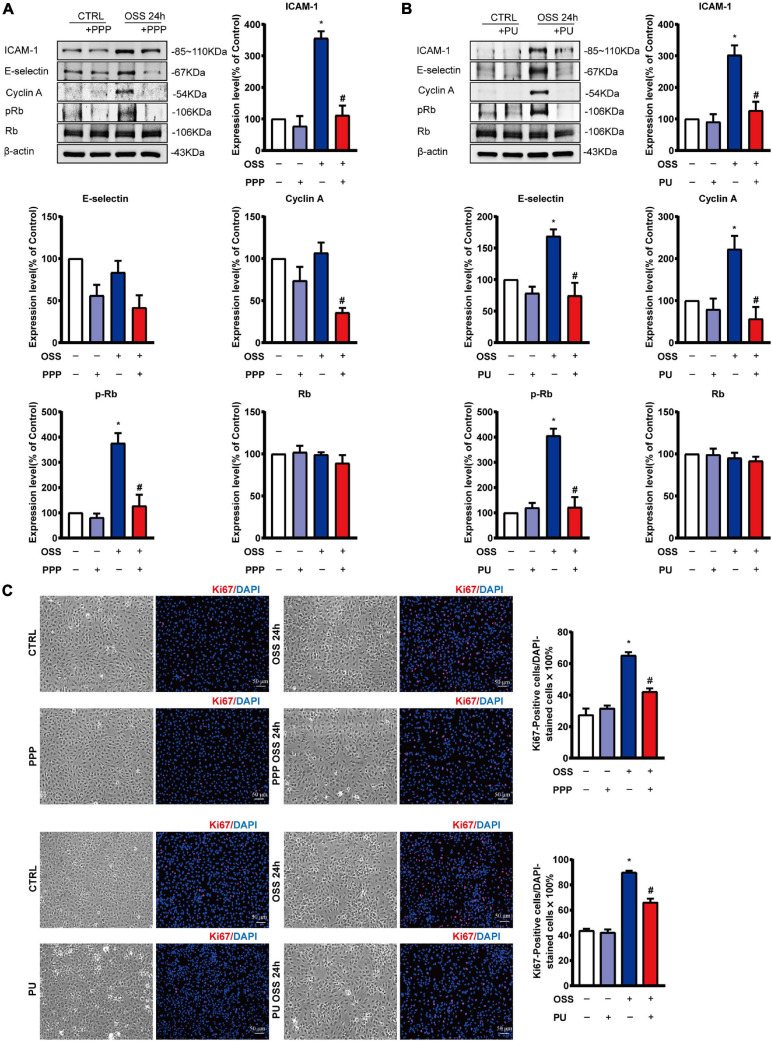
PPP and PU inhibit pro-inflammatory responses and proliferation of ECs induced by OSS. **(A,B)** Western blot analysis and quantitative results of the effects of PPP **(A)** or PU **(B)** on the expression of pro-inflammatory molecules ICAM-1, E-selectin and cell cycle regulatory proteins Cyclin A and pRb/Rb in ECs. Blot is a representative of five independent experiments. Ki67 immunostaining indicates that PPP/PU. **(C)** Inhibits OSS-induced EC proliferation (five independent experiments performed). *P < 0.05 vs. Vehicle, ^#^*P* < 0.05 vs. Model.

OSS-induced inflammation promotes proliferation of ECs. PPP or PU treatment significantly decreased protein expression of the pro-inflammatory molecules ICAM-1 and E-selectin in ECs after exposing to OSS for 24 h (Figures 7A,B). In addition, OSS-induced upregulation of cell cycle regulatory proteins in ECs, including Cyclin A and pRb, was all repressed by treating the ECs with either PPP or PU (Figures 7A,B). Results of immunofluorescence staining of Ki67, a marker of EC proliferation, showed that PPP or PU treatment significantly suppressed OSS-induced EC proliferation (Figure 7C). These data demonstrated that PPP and PU could inhibit force-specific activation of Smad1/5, which consequently attenuated pro-inflammatory responses and proliferation of ECs induced by disturbed flow with OSS.

### PPP and PU Inhibit TNF-α-Induced VSMC Migration, Phenotypic Modulation, and Pro-inflammatory Responses

**FIGURE 8:**
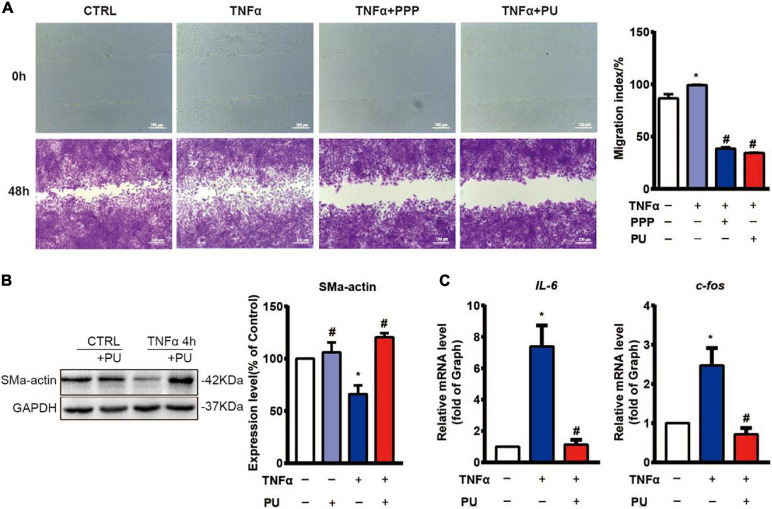
PPP and PU inhibit migration, phenotypic modulation and inflammation in VSMCs induced by TNF-α. **(A)** The migration of VSMCs was detected by wound-healing assay (three independent experiments performed). **(B)** Western blot analysis and quantitative results of the effects of PU on α-SMA expression in VSMCs. Blot is a representative of four independent experiments. **(C)** Real time PCR results on the IL-6 and c-fos mRNA expressions. Experiments were repeated five times independently. *P < 0.05, vs. Vehicle, ^#^*P* < 0.05 vs. Model.

Injured ECs and activated monocytes in plaques can release various growth factors, such as platelet-derived growth factor (PDGF), to promote migration and phenotypic modulation of VSMCs (Schachter, 1997). Since migration, proliferation, and phenotypic modulation of VSMCs are the critical factors contributing to the progression of atherosclerosis (Gomez and Owens, 2012), we first examined the effects of PPP and PU on TNF-α-induced migration of VSMCs using the wound-healing assay. The results showed that PPP and PU inhibited TNF-α-induced migration of VSMCs (Figure 8A). To further explore the effects of PU on phenotypic modulation of VSMCs, the gene expression of the contractile VSMC marker, i.e., SMα-actin, was determined (Figure 8B). The results showed that SMα-actin expression was reduced by TNF-α and this effect was reversed by PU treatment. The synthetic phenotype of VSMCs can release pro-inflammatory IL-6, and c-fos was shown to promote VSMC proliferation (Sylvester et al., 1998). We found that PU inhibited TNF-α-induced expression of IL-6 and c-fos in VSMCs (Figure 8C). Taken together, these results indicate that PPP and PU can protect against TNF-α-induced proliferation, migration, inflammation, and phenotypic modulation in VSMCs. All of these responses participate in the progression of atherosclerosis.

## Discussion

Accumulating evidence show that polyphenols from pomegranate fruit, juice and PE are beneficial to human health (Al-Jarallah et al., 2013; Kalaycioglu and Erim, 2017). Extracts from natural plants may have potential to be developed as new drugs for prevention and treatment of cardiovascular diseases (AlMatar et al., 2018).

Previous studies have shown that PE exerts an anti-atherogenic effect (Aviram et al., 2000; Kaplan et al., 2001; Rosenblat et al., 2006; Estrada-Luna et al., 2019), and lipid-lowering effect may account for part of its anti-atherogenic properties (Hou et al., 2019). In the present study, we used a mouse model of atherosclerosis through feeding LDLR^–/–^ mice with HFD for 12 weeks. The results showed that both PPP and PU can lower the lipids and reduce plaque formation. The effect of PU was greater than PPP, suggesting that PU is the effective anti-atherogenic agent in PPP. These results are consistent with the previous report showing that PE reduces plasma lipid levels in SR-B1/apoE double knockout mice (Al-Jarallah et al., 2013). In addition, other researchers reported that PE protects against atherosclerosis through inhibiting foam cell formation in the lesion areas (Aharoni et al., 2015; Bi et al., 2019). Our results provide the first line of evidence to show that treatment of macrophages with PPP or PU reduces ox-LDL uptake and inhibits LPS-induced pro-inflammatory responses, manifested by reduced mRNA expression of several pro-inflammatory factors, including IL-6, IL-1β, iNOS, MCP-1, and TNF-α.

Endothelial dysfunction is the initial step for the development of atherosclerosis. Disturbed flow with OSS is highly recognized to be the initial cause of EC dysfunction and pathogenesis of atherosclerosis (Buga et al., 1991; Lee et al., 2015). Under normal physiological condition, the hemodynamic forces stimulate NO release through expressing eNOS in ECs (Silacci et al., 2001), whereas eNOS activity is shown to be reduced at sites of perturbed shear stress (Go et al., 2014; Marchio et al., 2019). Previous studies showed that PE ameliorates perturbed shear stress-related atherosclerosis by regulating the expression of eNOS and oxidation-related genes in ECs (de Nigris et al., 2005; de Nigris et al., 2007). In addition, pomegranate juice was shown to enhance the biological actions of NO (Ignarro et al., 2006). Our results advanced the new notion to demonstrate that the vaso-protective effects of PPP and PU are endothelium-dependent. All these results suggest that the anti-atherogenic effects of PPP and PU may be attributable to their EC protective effects under disturbed flow with OSS.

Earlier studies suggest that laminar blood flow with PSS in the straight part of the arterial tree modulates cellular signaling and EC function, and is anti-atherogenic (Qin et al., 2007). By contrast, disturbed flow and its associated OSS in the branches and curvatures of the arterial tree promote EC dysfunction and thus aggravate atherosclerosis (Chiu and Chien, 2011). Our previous studies (Zhou et al., 2012, 2013) investigated the role of OSS in modulating EC mechanotransduction and hence the development of atherosclerosis, and demonstrated that EC layer expressed high levels of phosphorylation of BMPR-specific Smad 1/5 in the lesion areas exposed to disturbed flow with OSS. In this study we further generated an *in vivo* disturbed flow/OSS model in rats by using an U-shaped clip to constrict the rat abdominal aorta, and the results showed that high levels of phospho-Smad 1/5 were detected in the EC layer at post-stenotic regions of rat aortas, where disturbed flow with OSS occurred. PPP or PU administration significantly inhibited the OSS-induction of phospho-Smad 1/5 in the stenosed areas. *In vitro* studies on the ECs subjected to OSS also showed that PPP and PU attenuated OSS-induced Smad1/5 activation. In addition, PPP and PU could also reverse BMP-induced Smad1/5 activation. Our previous studies have demonstrated that disturbed flow with OSS can induce Smad1/5 phosphorylation through the sustained induction of bone morphogenetic protein receptor (BMPR) type IB (BMPRIB)-αvβ3 integrin association in ECs (Zhou et al., 2012, 2013). This OSS-induced sustained association of BMPRIB-αvβ3 integrin was mediated by the intracytoplasmic kinase domain of BMPRII and subsequently activated the Shc/focal adhesion kinase (FAK)/extracellular signal-regulated kinase (ERK) cascade, leading to Smad1/5 activation. Thus, it is possible that PPP and PU may share common pathways to exert regulatory effects on Smad1/5 activation through the BMPRII/BMPRIB-αvβ3 integrin signaling cascade in ECs in response to disturbed flow or cytokines/growth factors. The detailed mechanisms by which PPP and PU exert protective effects on ECs remain an interesting issue that warrants further investigation.

Previous studies indicated that OSS promotes inflammation and proliferation of vascular endothelium to aggravate EC dysfunction (Ajami et al., 2017; Sun et al., 2019). Application of OSS to ECs activated Smad 1/5 and led to up-regulation of cyclin A and down-regulation of p21 and p27 in ECs and hence EC proliferation (Zhou et al., 2012, 2013). Our results showed that PPP or PU treatment greatly retards cell cycle progression and proliferation of ECs. Besides, Gimbrone and Garcia-Cardena (2016) reported that OSS up-regulated the expression of adhesion molecules such as ICAM-1 and VCAM-1, which increased the recruitment of monocytes to endothelium, thus promoting vascular remodeling. Indeed, up-regulations of ICAM-1, VCAM-1, and E-selection in ECs after OSS exposure or TNF-α induction were also observed in the present study, and PPP or PU treatment inhibited the pro-inflammatory responses of ECs. We also found that the effect of PU on ICAM-1/VCAM-1 is more effective than PPP. This result is probably due to the reason that PU is a purified, effective compound of PPP, which is only a mixture. To sum up, our study advanced the new notion that treatment with PPP or PU can inhibit disturbed flow/OSS-induced activation of Smad1/5 and strongly suppressed the pro-inflammatory responses and proliferation of ECs induced by disturbed flow with OSS.

There is considerable evidence that laminar flow and associated shear stress significantly inhibit EC cell cycle progression and proliferation and enhance EC migration, and hence are atheroprotective. In contrast, disturbed flow with OSS can promote EC proliferation and inhibit EC migration, and hence is thought to be atherogenic (Chiu and Chien, 2011). Our previous studies also demonstrated that disturbed flow-induced activation of Smad1/5 can promote EC cell cycle progression and proliferation, which may contribute to the formation and progression of atherosclerosis (Zhou et al., 2012, 2013). In addition, there has been considerable evidence that disturbed flow with OSS or reduction in blood blow and shear stress can induce EC apoptosis and death (Chiu and Chien, 2011). Whether PPP/PU can modulate EC migration and exert protective effects on ECs to inhibit disturbed flow-induced EC apoptosis and death warrant further investigation.

In addition to ECs, VSMCs also play a vital role in maintaining vascular integrity and normal physiological function (Ross, 1975; Chistiakov et al., 2015; Lao et al., 2015). The aberrant interaction between ECs and VSMCs under pathological conditions promotes the occurrence and development of cardiovascular diseases, including atherosclerosis (Lao et al., 2015). It is widely accepted that VSMCs undergo phenotypic modulation during the progression of atherosclerosis, and the contractile phenotype of VSMCs converts to the synthetic phenotype, which triggers release of many pro-inflammatory factors, such as IL-6. Growth factors and inflammatory cytokines released from the injured ECs or other types of inflammatory cells in the plaques promote migration and pathological phenotypic modulation of VSMCs. Moreover, c-fos was shown to promote VSMC pathological proliferation (Hsieh et al., 1998; Sylvester et al., 1998; Fang et al., 2004). OSS-induced EC injury may promote abnormal activation of VSMCs, thereby accelerating pathological remodeling of blood vessels and promoting atherosclerosis. Here we reported for the first time that PU may inhibit migration, proliferation and phenotypic modulation of VSMCs induced by TNF-α, suggesting that PU has protective effects on VSMCs in response to inflammation. In addition to the direct effects of PPP/PU on VSMCs, it is also possible that the protective effects of PPP/PU on VSMCs might come from the anti-inflammatory effects of PPP/PU on ECs which may reduce the release of cytokines, such as TNF-α.

In summary, the present study demonstrates for the first time that PPP and PU protect EC dysfunction by inhibiting OSS-induced proliferation and inflammation. These protective effects of PPP and PU may be attributable to their inhibition of force-specific activation of Smad1/5 in ECs. Furthermore, the present results show that PPP and PU can inhibit inflammation, migration and phenotypic modulation of VSMCs under pro-inflammatory stimulation. Our findings provide new insights into the mechanisms by which PPP and PU inhibit disturbed flow/inflammation-induced EC dysfunction and VSMC proliferation, migration, and phenotypic modulation, with the consequent inhibition in atherosclerosis.

## Data Availability Statement

The original contributions presented in the study are included in the article/Supplementary Material, further inquiries can be directed to the corresponding author/s.

## Ethics Statement

The studies involving human participants were reviewed and approved by the experiments for the use of human umbilical cords were approved by the Hospital Human Subjects Review Committee (IRB Approval Nos. CGH-P101088 and CGH-CT9672) of Hsinchu Cathay General Hospital and were conducted under the guidelines established by the Ethics Review Board of National Health Research Institutes, Taiwan. Written informed consent was obtained from all individuals. The patients/participants provided their written informed consent to participate in this study. The animal study was reviewed and approved by This study was carried out in accordance with the principles of the Basel Declaration and Recommendations of Animal Care and Use Committee and PU IRB Laboratory Animal Welfare Committee in Peking University; the latter committee approved the protocol in this study (Approval No. LA2017193).

## Author Contributions

RQ and J-JC designed the study and revised the manuscript. X-LG and G-ZM prepared PPP extracts, purified PU, and analyzed the contents of PPP and PU by HPLC. Z-YC helped preparation and analysis of PPP and PU. GA and GL analyzed the data, prepared the figures, and wrote the manuscript. GL and W-LS performed *in vivo* studies. GA, C-IL, and P-LL performed *in vitro* experiments. Y-XW and X-YT performed endothelium-dependent vaso-protective assay. All authors read and approved the final manuscript.

## Conflict of Interest

The authors declare that the research was conducted in the absence of any commercial or financial relationships that could be construed as a potential conflict of interest.

## Abbreviations

OSS: oscillatory shear stress
PE: pomegranate extraction
PU: purified punicalagin
PPP: pomegranate peel polyphenols extraction
EC: endothelial cell
VSMC: vascular smooth muscle cell
HFD: high fat diet
BMPR: bone morphogenetic protein receptor
ICAM-1: intercellular adhesion molecule-1
VCAM-1: vascular cell adhesion molecule-1
TNF-α: tumor necrosis factor-α
MCP: monocyte chemotactic protein
TC: total cholesterol
TG: total triglyceride
iNOS: inducible nitric oxide synthase
eNOS: endothelial nitric oxide synthase
L-NAME: L-N (G)-nitroarginine methyl ester
IL-6: interleukin-6
HPLC: high performance liquid chromatography.
