# Supplementary material for: Punicalagin Attenuates Disturbed Flow-Induced Vascular Dysfunction by Inhibiting Force-Specific Activation of Smad1/5
Source: Front Cell Dev Biol. 2021 Jun 28;9:697539. doi: 10.3389/fcell.2021.697539 (PMC8273543; doi:10.3389/fcell.2021.697539)
Supplement: Supplementary file 1 [file Table_1.DOCX]

Supplementary Material

# Supplementary Method

## PPP extraction and PU purification

### Extraction of pomegranate peel polyphenol (PPP): All the pomegranate peels were pulverized and extracted by 60% ethanol. The obtained extraction was filtrated and concentrated under reduced pressure, and then separated and purified by column chromatography with microporous resin and with 50% ethanol as an eluant. The obtained eluant was concentrated and freeze-dried under vacuum to obtain PPP.

### Purification of punicalagin (PU) from PPP: The obtained PPP by column chromatography was separated and purified by gradient elution of ethanol at various concentrations, and the eluant of 10% ethanol was collected, concentrated, and freeze-dried under vacuum to obtain PU.

## Determination of punicalagin contents by HPLC

### Chromatographic conditions: chromatographic column: COSMOSIL Cholester (4.6 mm×250 mm, 5 μm); The mobile phase was comprised of A (methanol) and B (0.1% trifluoroacetic acid) . The gradient elution mode was set as follows: 0~10 min, 5% A:95% B; 10~25 min, 20% A:80% B; 25~35 min, 45% A:55% B; 35~45 min, 65% A:35% B. The flow rate was 1.0 mL/min, the column temperature was 30°C and the sample volume injected into the HPLC was 10 μL. Signal was detected at a wavelength of 370 nm.

### Determination of punicalagin contents: Punicalagin contents in the prepared PPP and PU samples were determined by an external standard method of HPLC under the conditions described as above. The punicalagin contents in the prepared samples were calculated by comparing the peak area with a punicalagin reference substance (purity 98%). As shown in Supplementary Fig 1 and Table S1, the retention time of the main chromatographic peaks of α- and β- punicalagin in the prepared PU sample (B) was consistent with that of punicalagin reference substance (A). The punicalagin contents in the prepared PPP and PU samples were determined and calculated as 42.08% and 90.6% , respectively.

## Plasma lipids analysis

After the mice were fasted for 4 h, their blood samples were drawn into tubes coated with heparin by retro-orbital venous plexus puncture and centrifuged at 1500 g for 10 min to collect plasma in supernatant. Plasma total cholesterol (TC) and triglycerides (TG) were measured according to the manufacturer's protocols (Sigma-Aldrich kits).

## Isolation and culture of primary macrophages

C57BL/6 mice weighting about 20 g were intraperitoneally injected with 4% Broth. Three days after injection, peritoneal lavage was performed using 10 mL of 0.01M phosphate-buffer saline (PBS), and peritoneal cells in the lavage were collected from each mouse. The cells at a concentration of 2×10^6^ cells/mL were pelleted and resuspended in RPMI 1640 cell culture medium with 10% FBS and 100 g/mL streptomycin and 100 IU/mL penicillin. Peritoneal macrophages were separated and planted in culture plates at 37 °C (5% CO_2_) for 4 h according to the literature ([Pineda-Torra *et al.*, 2015](#_ENREF_34)). The peritoneal macrophages cultured in 6-well palates were incubated with 50 µg/mL ox-LDL for 24 h or 1 µg/mL LPS for 12 h to induce polarization or inflammation, respectively.

## Foam cell staining

The peritoneal macrophages cultured in 12-well palates were pre-incubated for 24 h with ox-LDL (50 µg/mL). The cells were fixed with 4% paraformaldehyde and then washed with 0.01 M PBS and stained with oil red O at 37 °C for 30 min and cell morphology was observed.

## Statistical analysis

All data are expressed as mean ± standard error of the mean (SEM). Differences between mean values of normally distributed data were analyzed using one-way ANOVA (Dunnett’s t test) and two tailed Student’s t test. P<0.05 was considered to indicate a statistically significant difference.

# Supplementary Figures and Tables

## Supplementary Figures

**Supplementary Figure 1.** **HPLC chromatogram of the** **punicalagin reference substance and** **the prepared PU samples.** A: punicalagin reference substance; B: the prepared PU samples. Punicalagin has two isoforms of α and β (1: α- punicalagin, 2: β- punicalagin), and punicalagin content is calculated by the ratio of the peak areas of α and β isoforms to the total peak areas.


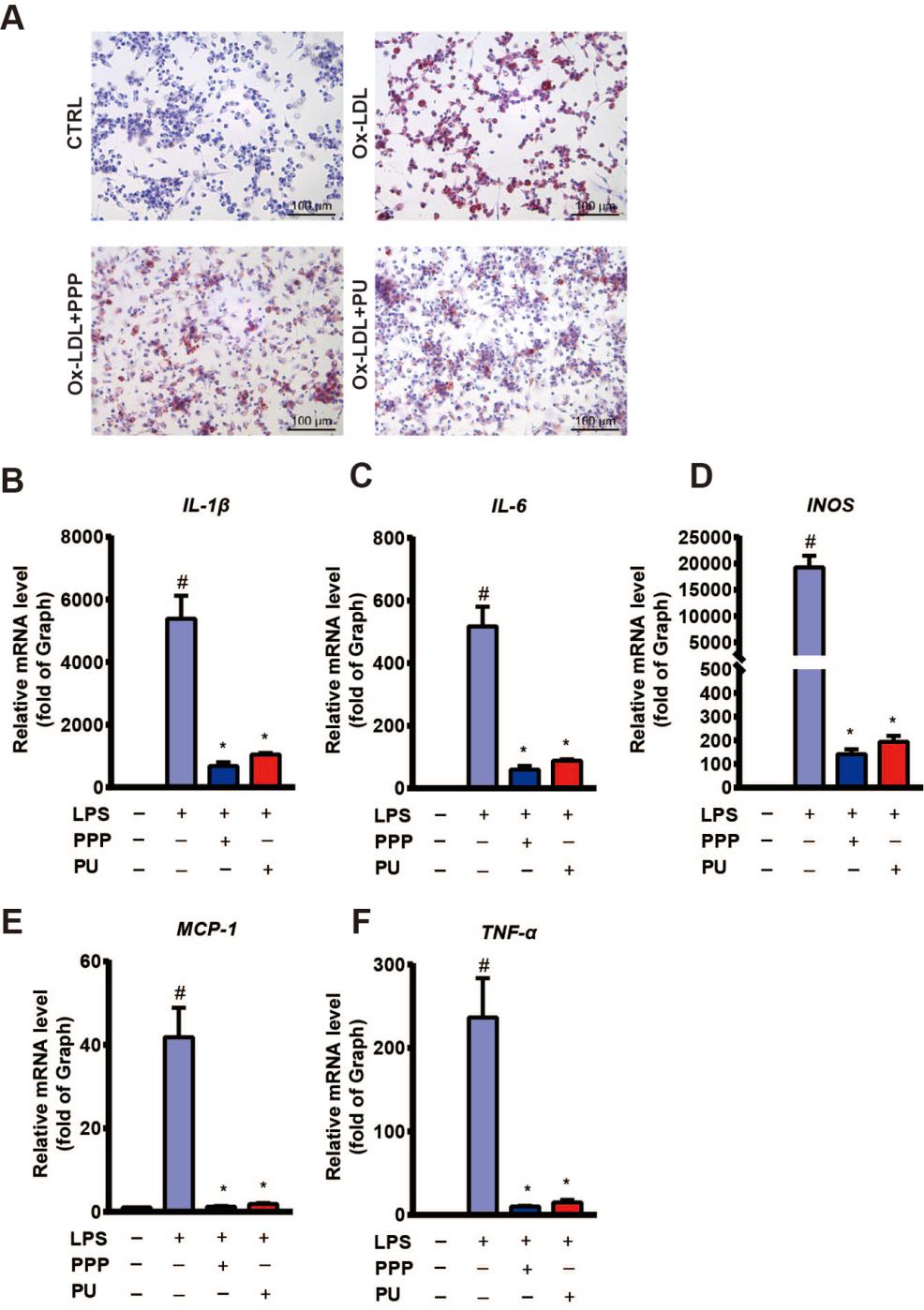


**Supplementary Figure 2.** **PPP and PU inhibit ox-LDL-induced foam cell formation and LPS-induced inflammation in macrophages.** A. Oil-red O staining indicates that PPP and PU inhibit ox-LDL-induced lipid uptake in macrophages; B-F. real-time PCR analysis of PPP and PU on gene expression of pro-inflammatory cytokines of IL-1b, IL-6, TNF-a, INOS, MCP-1 and quantitative results. *P<0.05 *vs* Vehicle, ^#^P<0.05 *vs* Model.


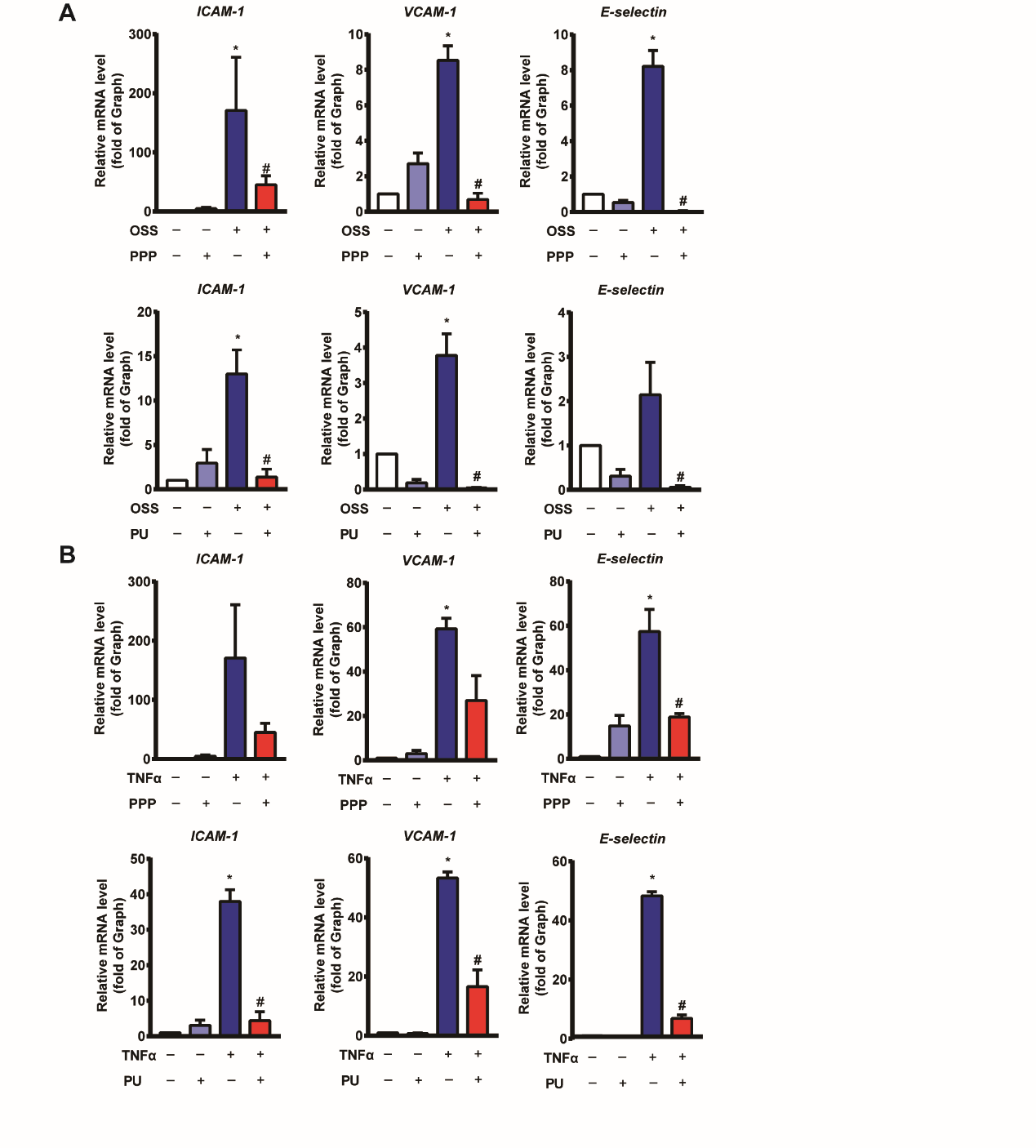


**Supplementary Figure 3.** **PPP and PU inhibit OSS-induced inflammatory response of ECs.** A-B. PCR analysis of the mRNA expressions of the indicated molecules in ECs induced by OSS (A) or TNF-α (B) stimulation. *P<0.05 *vs* Vehicle, ^#^P<0.05 *vs* Model.

## Supplementary Tables

**Supplementary Table S1. Punicalagin contents in the prepared samples**

| No | Sample mass (mg) | Punicalagin mass in the sample (mg) | Punicalagin content in the sample (%) |
| --- | --- | --- | --- |
| PPP | 23.40 | 9.846 | 42.08 |
| PU | 62.12 | 56.28 | 90.6 |

**Supplementary Table S2. List of primers for gene cloning or real-time PCR**

| Primer* | Sequences (5`-3`) | NCBI Reference Sequence |
| --- | --- | --- |
| hICAM-1-F | ATGCCCAGACATCTGTGTCC | NM_000201.3 |
| hICAM-1-R | GGGGTCTCTATGCCCAACAA |  |
| hVCAM-1-F | GGGAAGATGGTCGTGATCCTT | NM_001199834 |
| hVCAM-1-R | TCTGGGGTGGTCTCGATTTTA |  |
| hE-selectin-F | AGAGTGGAGCCTGGTCTTACA | NM_000450 |
| hE-selectin-R | CCTTTGCTGACAATAAGCACTGG |  |
| rIL-6-F | GGGATCTGGACAAGTCATCCC | NM_012589 |
| rIL-6-R | GGGTATCTATCTGCTGCCACT |  |
| rc-fos-F | CGGGTTTCAACGCCGACTA | NM_022197.2 |
| rc-fos-R | TTGGCACTAGAGACGGACAGA |  |
| miNOS-F | GTTCTCAGCCCAACAATACAAGA | NM_010927 |
| miNOS-R | GTGGACGGGTCGATGTCAC |  |
| mIL-1β-F | GCAACTGTTCCTGAACTCAACT | NM_008361 |
| mIL-1β-R | ATCTTTTGGGGTCCGTCAACT |  |
| mTNF-α-F | CCCTCACACTCAGATCATCTTCT | NM_013693 |
| mTNF-α-R | GCTACGACGTGGGCTACAG |  |
| mMCP-1-F | TTAAAAACCTGGATCGGAACCAA | NM_011333 |
| mMCP-1-R | GCATTAGCTTCAGATTTACGGGT |  |
